# Mr^2^DNM: A Novel Mutual Information-Based Dendritic Neuron Model

**DOI:** 10.1155/2019/7362931

**Published:** 2019-08-01

**Authors:** Xiaoxiao Qian, Yirui Wang, Shuyang Cao, Yuki Todo, Shangce Gao

**Affiliations:** ^1^Faculty of Engineering, University of Toyama, Toyama-shi 930-8555, Japan; ^2^College of Civil Engineering, Tongji University, Shanghai 200092, China; ^3^School of Electrical and Computer Engineering, Kanazawa University, Kanazawa-shi 920-1192, Japan

## Abstract

By employing a neuron plasticity mechanism, the original dendritic neuron model (DNM) has been succeeded in the classification tasks with not only an encouraging accuracy but also a simple learning rule. However, the data collected in real world contain a lot of redundancy, which causes the process of analyzing data by DNM become complicated and time-consuming. This paper proposes a reliable hybrid model which combines a maximum relevance minimum redundancy (Mr^2^) feature selection technique with DNM (namely, Mr^2^DNM) for classifying the practical classification problems. The mutual information-based Mr^2^ is applied to evaluate and rank the most informative and discriminative features for the given dataset. The obtained optimal feature subset is used to train and test the DNM for classifying five different problems arisen from medical, physical, and social scenarios. Experimental results suggest that the proposed Mr^2^DNM outperforms DNM and other six classification algorithms in terms of accuracy and computational efficiency.

## 1. Introduction

As a machine learning technique, a supervised learning algorithm is usually evaluated with a dataset which includes training samples and testing samples. Each sample is depicted by a certain number of features (or attributes) and a class label, e.g., for the medical diagnosis, the features might consist of the age, sex, and smoking habit of a patient, and the class label is the corresponding diagnosis result that the patient is whether or not suffering from liver disorders [1]. After learning, the classifier can obtain learning rules that can be applied to classify future samples in the same domain. However, most domains are explored with less than 40 features before 1997 [2]. It should not be tolerated that the dimension issue of the dataset leads the study to only explore on a limited scale. To explore the domains with more features, the optimization of the dataset is urgent and challenging. Regarding the feature of a dataset, the concept of “relevance” is firstly proposed by John et al. [3] in the context of machine learning. That motivates Langley [4] to develop a relevant features selection method for assisting the learning of the classifier. However, selecting the most relevant feature through finding or ranking all the relevant features of the dataset is generally suboptimal for training a classifier, especially if the features include duplicate information, which is called redundant feature. Therefore, a maximum relevance minimum redundancy (Mr^2^) feature selection framework that can eliminate most irrelevant and redundant features to reduce training samples is proposed for gene expression array analysis [5]. Generally, in a gene expression dataset which contains 6,000∼60,000 samples, there are only less than 100 samples which are suitable for training and testing. Hence, the feature selection provides a good solution for developing the gene domain. The objective of the feature selection is to avoid the curse of dimensionality of the dataset and thereafter to improve the classification performance of the classifiers. It can not only provide better classification accuracy with lower computation cost, but also give an easier understanding of the importance of the feature in the dataset. The feature selection methods have driven the classifier to explore more domains; particularly, those consist of numerous features. It has been widely applied to areas of text processing of Internet documents [6], combinatorial chemistry [2], etc.

To achieve the best performance of classification, in addition to the feature selection, the classifier is another crucial factor. Among hundreds of classifiers, the artificial neural networks (ANNs) occupy an important place. ANNs are inspired by biological systems with lots of interconnected simple processors [7, 8] and are widely applied for solving problems arisen from many different fields, e.g., business, industry, and science [9]. The well-known mathematical neuron model called McCulloch–Pitts model (MCP) [10] defines the corresponding weights for the synapses to control the importance of the inputs. In recent years, many studies [11–13] suggest that the information processing capacity-based MCP of a single neuron has not been fully developed. As the MCP-based single neuron model is too oversimplified to address nonlinearly separated problems [14, 15], it is considered that the utilization of the dendritic structure [16, 17] is promising to improve the nonlinear processing ability for a neuron. Although the Koch–Poggio–Torre model [18] considers the effects of dendrites in the neuron, it lacks the plasticity mechanism, that is, the synaptic type and dendritic structure cannot correctly classify some complex tasks [19]. Some studies [20–23] have pointed out that some pyramidal neurons possess the plasticity mechanism, which might provide inspirations for improving the Koch–Poggio–Torre model.

In our previous works, we mainly focus on the development of a single dendritic neuron model (DNM) via the nonlinear information processing ability of synapses [24]. DNM has been applied to medical diagnosis [25, 26], tourism prediction [27, 28], and financial time series prediction [29]. Besides its supervised learning ability, an unsupervised learnable DNM has been used for efficiently learning the two-dimensional multidirectional selectivity problem [30]. In addition, DNM trained by six population-based evolutionary learning algorithms also shows its prominent effects in classification, approximation, and prediction [31]. In DNM, the neuron plasticity mechanism is realized by synaptic pruning and dendritic pruning during learning. Meanwhile, the obtained simplified morphological of DNM can be implemented with hardware logical circuits [32].

To reduce the influence of redundancy feature on the dataset and save computation cost, in this paper we propose a hybrid model Mr^2^DNM by combining Mr^2^ with DNM. Mr^2^DNM applies an optimal subset to train and generate learning rules, where the optimal subset is obtained by utilizing Mr^2^ criteria to search and rank the features of the dataset, and DNM is used to evaluate the subset. Meanwhile, the unused samples of the optimal subset will be used as testing ones to verify the performance of Mr^2^DNM. In the experiment, the proposed model is compared with other six classification models by classifying five real-world benchmark datasets, which includes three well-known medical diagnosis datasets (i.e., breast cancer, liver disorders, and diabetes), one radar dataset that returns from the ionosphere, and one congressional voting records dataset. Results suggest that the proposed model outperforms its peers in terms of the classification accuracy, computational efficiency, convergence rate, and the quality of the area under the receiver operator characteristic (ROC) curve.

The remaining of this paper is organized as follows.  Section 2 presents a brief introduction of the fundamental structures and functions of Mr^2^DNM.  Section 3 introduces the error back-propagation learning algorithm that is applied to train Mr^2^DNM.  Section 4 shows the experimental results of the model and performance analysis on five benchmark datasets. Finally, the conclusions are drawn in Section 5.

## 2. Proposed Model: Mr^2^DNM

### 2.1. Mr^2^

**Figure 1:**
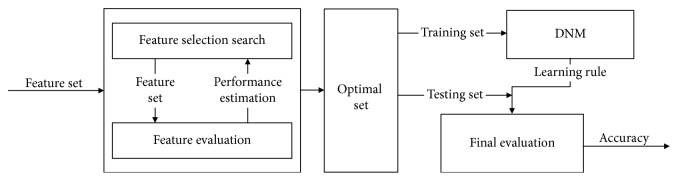
The wrapper approach to the proposed Mr^2^DNM.

The proposed Mr^2^DNM is a hybrid approach based on a feature selection technique and a neural network classifier, which are combined using a wrapper approach as shown in Figure 1. The feature selection is implemented via the criteria of Mr^2^ based on mutual information. By calculating the mutual information of dataset, relevances of (1) feature-feature and (2) feature-target class are visually quantified. Furthermore, information overlap between features (i.e., feature-feature) is considered and defined as redundancy. The feature subset which is obtained by Mr^2^ criteria includes ordered (strongly ⟶ weakly) relevance features. The relevance of the feature decides the frequency of the feature joining into the learning process of a classifier (i.e., strongly—always ⟶ weakly—possibly). Meanwhile, the irrelevant features are excluded from the optimal feature subset during the learning of the classifier. Therefore, Mr^2^ feature selection combining with plasticity neurons of DNM is supposed to reduce the computational burden (e.g., learning process acceleration), avoid the overfitting problem, and enhance the generalization capacity of Mr^2^DNM [33–35]. The Mr^2^ criterion based on mutual information [34] is expressed as follows:

(1) $$\begin{array}{c} \max\Phi \left(D,R\right),\Phi =D-R, \end{array}$$

where *D* represents the maximal relevance of a feature set *S* with *N* features *x*_*i*_. Φ(·) expresses the optimize operation which combines *D* and *R* to find an optimal feature subset. The equation of *D* is defined as

(2) $$\begin{array}{c} \max\;\;D=\frac{1}{\left|S\right|}\underset{x_{i}\in S}{\sum }I\left(x_{i};c\right),\;i=1,\ldots ,n, \end{array}$$

where *I* represents the mutual information between individual feature *x*_*i*_ ∈ *S* and the target class *c*. In addition, it is considered that there is redundancy in two highly dependent features. In this case, one of the two features can be removed and it will not influence the discriminative power [33]. Therefore, *R* is used to compute the minimal redundancy of a feature set *S*, shown as

(3) $$\begin{array}{c} \min R=\frac{1}{{\left|S\right|}^{2}}\underset{x_{i},x_{r}\in S}{\sum }I\left(x_{i};x_{r}\right),\;i,r=1,\ldots ,n, \end{array}$$

where the mutual information *I*(*x*; *y*) of two random variables *x* and *y* can be expressed in terms of their probabilistic density (or distribution) functions *p*(*x*), *p*(*y*), and *p*(*x*, *y*), for continuous (or discrete) case

(4) $$\begin{array}{c} I\left(x;y\right)=\iint p\left(x,y\right)\log\left(\frac{p\left(x,y\right)}{p\left(x\right)p\left(y\right)}\right)dx\;dy,\\ I\left(x;y\right)=\underset{y\in Y}{\sum }\underset{x\in X}{\sum }p\left(x,y\right)\log\left(\frac{p\left(x,y\right)}{p\left(x\right)p\left(y\right)}\right). \end{array}$$

In the Mr^2^ criterion, the ranking of all *N* features *X*=*x*_*i*_{*i*=1,…, *N*} in the dataset is done via selecting the features with the maximal Φ(·) in turn. Among them, the near-optimal features defined by Φ(·) can be found with an incremental search method [34]. The incremental search method is defined as follows:

(5) $$\begin{array}{c} \underset{x_{r}\in \left\{X-S_{n-1}\right\}}{\max}\left[I\left(x_{r};c\right)-\frac{1}{n-1}\underset{x_{i}\in S_{n-1}}{\sum }I\left(x_{r};x_{i}\right)\right], \end{array}$$

where *S*_*n*−1_ is the feature set with *n* − 1 features. The task of this incremental search method is to select the *n*th feature from the set {*X* − *S*_*n*−1_}. The computational complexity of the incremental search method is *O*(|*S*| · *N*).

Additionally, the features are defined as *F*_1_(*i*_1_), *F*_2_(*i*_2_),…, *F*_*N*_(*i*_*N*_), where *F*_*N*_ represents the given mark of the feature in the dataset, *i*_*N*_ is the ranking of the feature which is obtained by the Mr^2^ criterion, and for example, *i*_*N*_=1 indicates that the feature *F*_*N*_ ranks the first one in the dataset and should be the most important feature, which has the maximal relevance with the target class *c* and the minimal redundancy in comparison with the other features, while *i*_*N*_=*N* means the feature *F*_*N*_ can be firstly excluded from the learning of the classifier to speed up the calculation efficiency. The DNM combines with the ranked features to achieve the optimal compromised solution between classification accuracy rate and dataset dimension.

### 2.2. DNM

In DNM, the dendrites and synapses are formed via initial user-defined parameters in the primary neuron system. The initial structure is allowed to possess superfluous number of dendrites and synapses. The superfluous parts are screened; meanwhile, the useful parts are strengthened and fixed to form the ripened structure of the neuron model during learning. Four basic rules are used to define the DNM, shown as follows:

- The model allows initial number of dendrites and synapses which can be arbitrarily defined.
- The interaction exists among all synapses in the same dendrite layer.
- The ripened dendrites and synapses are decided by learning.
- The synapses can only be defined as one of the four specific connection states.

**Figure 2:**
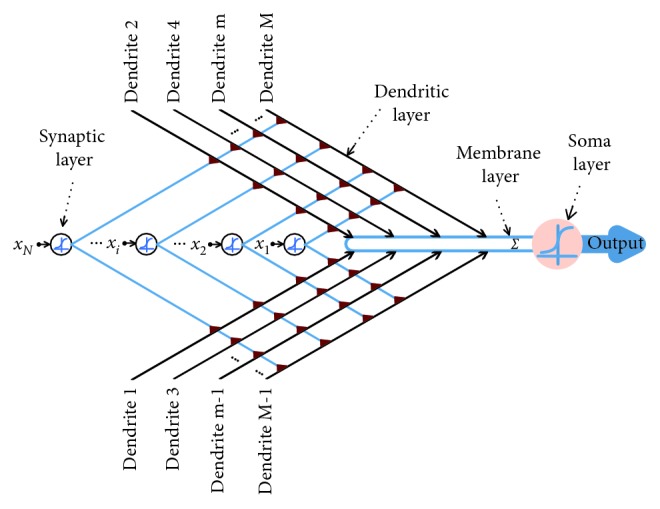
The structure of the DNM.

In Figure 2, the transmission process of signals in the model during learning is illustrated. It can be summarized as follows:

- The input signals for one specific task are transferred to synapses via sigmoid functions and output to dendritic branches.
- The results from synapses on the same dendritic branch are calculated by applying a multiplication operation.
- The signals from all dendritic branches are collected in the membrane layer and summed to the soma layer.
- The signal is determined in the soma layer whether it exceeds the threshold or not.

#### 2.2.1. Synaptic Layer

**Figure 3:**
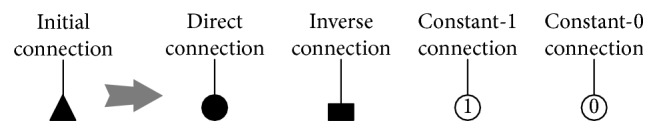
Four connection types in the synaptic layer.

A synapse is produced by the contact of two neurons. Its duty is to transmit information within two neurons. In the synaptic layer of our model, the synapse can be defined as the specific one of the four connection types, while as an input to interact with the dendritic branch. The four connection types include the direct connection, inverse connection, constant-0 connection, and constant-1 connection, which can be expressed by sigmoid functions. The four connection types are illustrated in Figure 3. The changes in the postsynaptic potential caused by ion can be used to decide whether the input is an excitation synapse or an inhibition one [36]. The node function that connecting *i*th (*i*=1,2,3,…, *N*) input to the *j*th (*j*=1,2,3,…, *M*) synaptic layer is expressed as follows:

(6) $$\begin{array}{c} Y_{i,j}=\frac{1}{1+e^{-k\left(\omega_{i,j}x_{i}-q_{i,j}\right)}}, \end{array}$$

where *Y*_*i*,*j*_ indicates the output of the synaptic layer. *x*_*i*_ ∈ [0,  1] denotes the input of the synapse. *k* represents a user-defined parameter, whose optimal setting will be given in the experiment. The weight parameters *ω*_*i*,*j*_ and *q*_*i*,*j*_ in the synapses need to be trained by learning algorithms. The following equation is used to compute the threshold *θ*_*i*,*j*_ of the synaptic layer:

(7) $$\begin{array}{c} \theta_{i,j}=\frac{q_{i,j}}{\omega_{i,j}}. \end{array}$$

**Figure 4:**
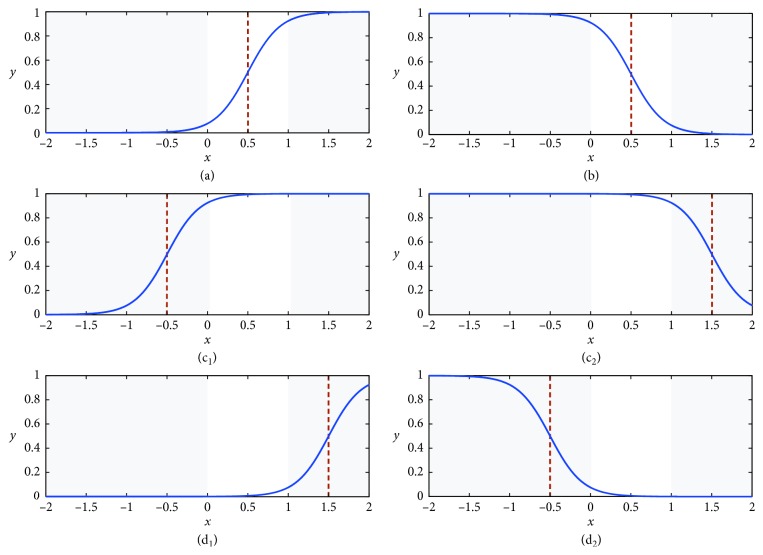
Six function cases of the synaptic layer. (a) Direct connection. (b) Inverse connection. (c1) Constant-1 connection. (c2) Constant-1 connection. (d1) Constant-0 connection. (d2) Constant-0 connection.

The presynaptic input is determined as one of the four connection types via the trained *ω*_*i*,*j*_ and *q*_*i*,*j*_ values. The details of these four connection types are shown in Figure 4, and the functions of six cases are given as follows:

- Type 1: direct connection
- Case (a): 0 < *q*_*i*,*j*_ < *ω*_*i*,*j*_, e.g., *ω*_*i*,*j*_=1.0 and *q*_*i*,*j*_=0.5.

(8) $$\begin{array}{c} Y_{i,j}=\left\{\begin{array}{cc} 1, & \;\text{if }x_{i}>\theta_{i,j},\\ 0, & \;\text{if }x_{i}\leq \theta_{i,j}. \end{array}\right. \end{array}$$

  In this case (Figure 4(a)), when the input *x*_*i*_ value exceeds the threshold *θ*_*i*,*j*_, the output *Y*_*i*,*j*_ is 1, which means the signals will be passed and output smoothly. Otherwise, the signals will be blocked.
(ii) Type 2: inverse connection
  Case (b): *ω*_*i*,*j*_ < *q*_*i*,*j*_ < 0, e.g., *ω*_*i*,*j*_=−1.0 and *q*_*i*,*j*_=−0.5.

(9) $$\begin{array}{c} Y_{i,j}=\left\{\begin{array}{cc} 0, & \;\text{if }x_{i}>\theta_{i,j},\\ 1, & \;\text{if }x_{i}\leq \theta_{i,j}, \end{array}\right. \end{array}$$

where the threshold *θ*_*i*,*j*_ is not exceeded by the input *x*_*i*_ value, the output *Y*_*i*,*j*_ is 1, which means the signal is updated as an excitatory signal and allows the information to pass, shown in Figure 4(b). The inverse connection type is considered as a logic NOT operation.

(iii) Type 3: constant-1 connection
  Case (c_1_): *q*_*i*,*j*_ < 0 < *ω*_*i*,*j*_, e.g., *ω*_*i*,*j*_=1.0 and *q*_*i*,*j*_=−0.5;
  Case (c_2_): *q*_*i*,*j*_ < *ω*_*i*,*j*_ < 0, e.g., *ω*_*i*,*j*_=−1.0 and *q*_*i*,*j*_=−1.5.
  In the constant-1 connection cases (Figures 4(c1) and 4(c2)), the outputs are always 1, regardless of the inputs or the parameters change. The information will be transmitted completely.
(iv) Type 4: constant-0 connection
  Case (d_1_): 0 < *ω*_*i*,*j*_ < *q*_*i*,*j*_, e.g., *ω*_*i*,*j*_=1.0 and *q*_*i*,*j*_=1.5;
  Case (d_2_): *ω*_*i*,*j*_ < 0 < *q*_*i*,*j*_, e.g., *ω*_*i*,*j*_=−1.0 and *q*_*i*,*j*_=0.5.
  In the two cases (Figures 4(d1) and 4(d2)) which are contrasted to the constant-1 connection cases, all the information will be blocked; in other words, the input values can be ignored.

The weight parameters *ω*_*i*,*j*_ and *q*_*i*,*j*_ are assigned with random values from −1.5 to 1.5, before the model begins the training. Therefore, the synaptic types are also the random connection types. When the model finishes the training and generates the learning rule, the model obtains the correct weight parameters *ω*_*i*,*j*_ and *q*_*i*,*j*_. Then the synaptic connection types can be determined.

#### 2.2.2. Dendritic Layer

The dendritic layer receives the signals from the synaptic layers and implements a multiplication operation. The multiplication operation approximately corresponds to a logical AND operation and is described by

(10) $$\begin{array}{c} Z_{j}=\overset{N}{\underset{i=1}{\prod }}Y_{i,j}. \end{array}$$

#### 2.2.3. Membrane Layer

The signals that come from the dendritic branch are summed in the membrane layer. This summation is approximately equal to a logical OR operation and is expressed as follows:

(11) $$\begin{array}{c} V=\overset{M}{\underset{j=1}{\sum }}Z_{j}. \end{array}$$

#### 2.2.4. Soma Layer

The soma layer is the last step of a neuronal computation and associated with a threshold. If the signal from the membrane exceeds the threshold, the transmission channel is turned on. The operation is defined as a sigmoid function and is shown as follows:

(12) $$\begin{array}{c} O=\frac{1}{1+e^{-k_{\text{soma}}\left(V-\theta_{\text{soma}}\right)}}, \end{array}$$

where *k*_soma_ is a user-defined parameter, *θ*_soma_ means the threshold of the cell body and its range is [0,1]. When the signal from the membrane layer is greater than the threshold, the neuron excitation will occur, otherwise keep fired.

#### 2.2.5. Neuronal Pruning Function

The neuronal pruning functions in the synaptic layer and dendritic layer complete the plasticity mechanism of the proposed model. Based on classification problems, the proposed model can give the specific pruning structure by applying the synaptic pruning and dendritic pruning.

*(1) Synaptic Pruning*. The constant-1 synaptic connection in the four connection types is considered as one of the origins of the plasticity of the neuron, which is called the synaptic pruning. The constant-1 completes a multiplication operation in the dendritic layer, since every synapse interacts with the other synapses in each dendritic layer. A value multiplied by the constant-1 is not changed, and it does not cause the output of the dendritic layer to change. Therefore, this constant-1 synaptic connection type can be neglected or pruned in the dendritic layer to simplify the neuron model without having any impact on the learning process of the proposed model.

*(2) Dendritic Pruning*. The constant-0 synaptic connection interacts with each dendritic layer, which is called dendritic pruning. Hence, whatever the output of the dendritic layer is, it multiplied by the constant-0 always equals 0. The outputs of all the dendritic layers are summed in the membrane layer, and any value that adds zero is equal to itself. The corresponding dendrite with constant-0 can be removed without any impact, which can simplify the morphology and structure of the proposed model.

## 3. Learning Algorithm

Based on the structure of the proposed Mr^2^DNM which is a feed-forward logic neural network, the error back-propagation (BP) algorithm is employed for training the model. The construction of the neuron model depends on an effective learning rule. Its learning rule is obtained by the least squared error between the real output vector *O* and the target output vector *T*, shown as follows:

(13) $$\begin{array}{c} E=\frac{1}{2}{\left(T-O\right)}^{2}. \end{array}$$

The error is decreased by correcting the synaptic parameters *ω*_*i*,*j*_ and *q*_*i*,*j*_ of the connection function during learning. The corrections of both parameters utilize the gradient descent learning algorithm. The equations are expressed as follows:

(14) $$\begin{array}{c} \Delta \omega_{i,j}\left(t\right)=-\eta \frac{\partial E}{\partial \omega_{i,j}},\\ \Delta q_{i,j}\left(t\right)=-\eta \frac{\partial E}{\partial q_{i,j}}, \end{array}$$

where *η* represents the learning rate, which is a user-defined parameter. However, a small learning rate might make the convergence speed slow. Thus, we set the corresponding suitable *η* for each classification problem as possible in the simulation. Then, the updating rules of *ω*_*i*,*j*_ and *q*_*i*,*j*_ are computed as follows:

(15) $$\begin{array}{c} \omega_{i,j}\left(t+1\right)=\omega_{i,j}\left(t\right)+\Delta \omega_{i,j},\\ q_{i,j}\left(t+1\right)=q_{i,j}\left(t\right)+\Delta q_{i,j}, \end{array}$$

where *t* is the number of the learning iteration. In addition, the partial differentials of *E* with regard to *ω*_*i*,*j*_ and *q*_*i*,*j*_ are defined as follows:

(16) $$\begin{array}{c} \frac{\partial E}{\partial \omega_{i,j}}=\frac{\partial E}{\partial O}\cdot \frac{\partial O}{\partial V}\cdot \frac{\partial V}{\partial Z_{j}}\cdot \frac{\partial Z_{j}}{\partial Y_{i,j}}\cdot \frac{\partial Y_{i,j}}{\partial \omega_{i,j}},\\ \frac{\partial E}{\partial q_{i,j}}=\frac{\partial E}{\partial O}\cdot \frac{\partial O}{\partial V}\cdot \frac{\partial V}{\partial Z_{j}}\cdot \frac{\partial Z_{j}}{\partial Y_{i,j}}\cdot \frac{\partial Y_{i,j}}{\partial q_{i,j}}. \end{array}$$

The detail parts of the above partial differentials are represented as follows:

(17) $$\begin{array}{c} \frac{\partial E}{\partial O}=O-T,\\ \frac{\partial O}{\partial V}=\frac{k_{soma}e^{-k_{soma}\left(V-q_{soma}\right)}}{{\left(1+e^{-k_{soma}\left(V-q_{soma}\right)}\right)}^{2}},\\ \frac{\partial V}{\partial Z_{j}}=1,\\ \frac{\partial Z_{j}}{\partial Y_{i,j}}=\overset{N}{\underset{L=1\&L\neq i}{\prod }}Y_{L,j},\\ \frac{\partial Y_{i,j}}{\partial w_{i,j}}=\frac{kx_{i}e^{-k\left(x_{i}w_{i,j}-q_{i,j}\right)}}{{\left(1+e^{-k\left(x_{i}w_{i,j}-q_{i,j}\right)}\right)}^{2}},\\ \frac{\partial Y_{i,j}}{\partial q_{i,j}}=\frac{-ke^{-k\left(x_{i}w_{i,j}-q_{i,j}\right)}}{{\left(1+e^{-k\left(x_{i}w_{i,j}-q_{i,j}\right)}\right)}^{2}}. \end{array}$$

## 4. Experiment and Analysis

### 4.1. Experimental Setup

This experiment is programmed in MATLAB (R2013b) and implemented on a computer with Intel(R) Core i5 3.4 GHz and RAM 16 GB. To assess the performance of the proposed Mr^2^DNM, five widely used benchmark datasets taken from the University of California at Irvine Machine Learning Repository (UCI) are tested [37]. These datasets include Wisconsin breast cancer database (WBCD), BUPA medical research database for liver disorders (BUPA), ionosphere dataset (IONO), Pima Indians diabetes dataset (PIMA), and congressional voting records dataset (VOTE). These five datasets could be divided into categorical (WBCD, BUPA) or numerical (IONO, PIMA, VOTE) ones.  Table 1 lists the characteristics of these datasets. To make a fair comparison, the samples which include missing value are deleted, because the used classifiers cannot handle missing value. According to our previous work, the samples of each dataset are randomly divided: 70% for training and 30% for testing [26]. In addition, the input variables are normalized from 0 to 1.0, by a min-max normalization rule:

(18) $$\begin{array}{c} X_{\text{normalized}}=\frac{X-X_{\min}}{X_{\max}-X_{\min}}. \end{array}$$

Table 2 provides the user-defined parameter settings to our experiment for each dataset independently. Among them, the parameter settings of five datasets are set based on the suggesting in [25, 26].

### 4.2. Performance Evaluation

**Figure 5:**
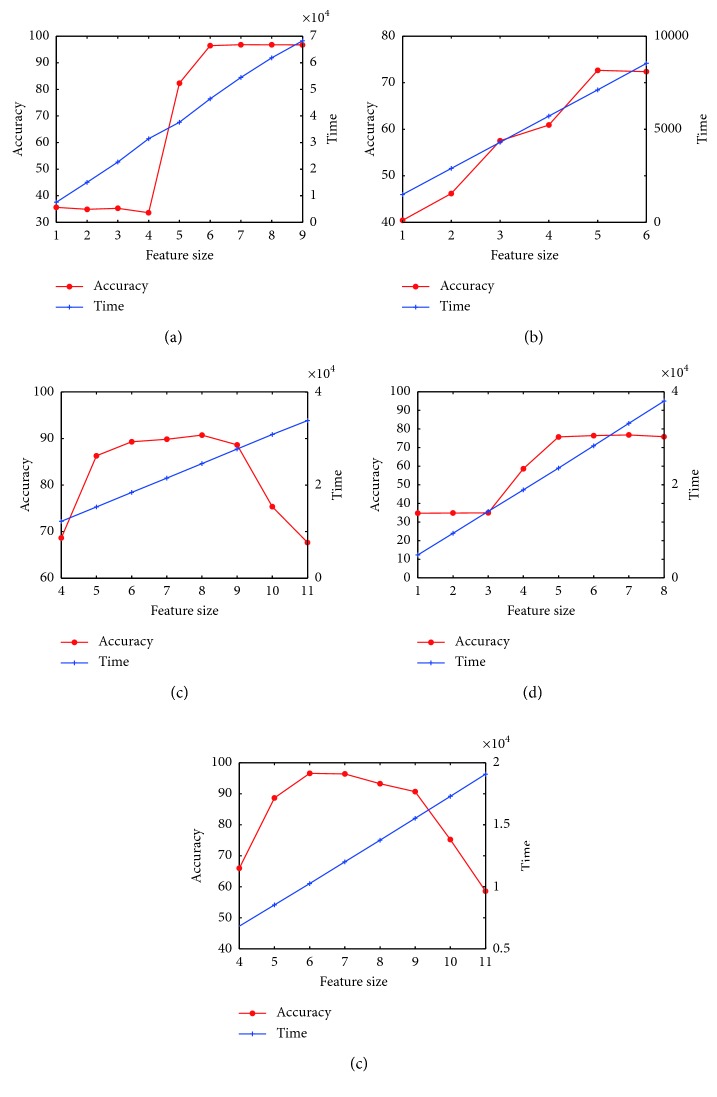
Accuracy, time, and feature size for five datasets. (a) WBCD. (b) BUPA. (c) IONA. (d) PIMA. (e) VOTE.

**Figure 6:**
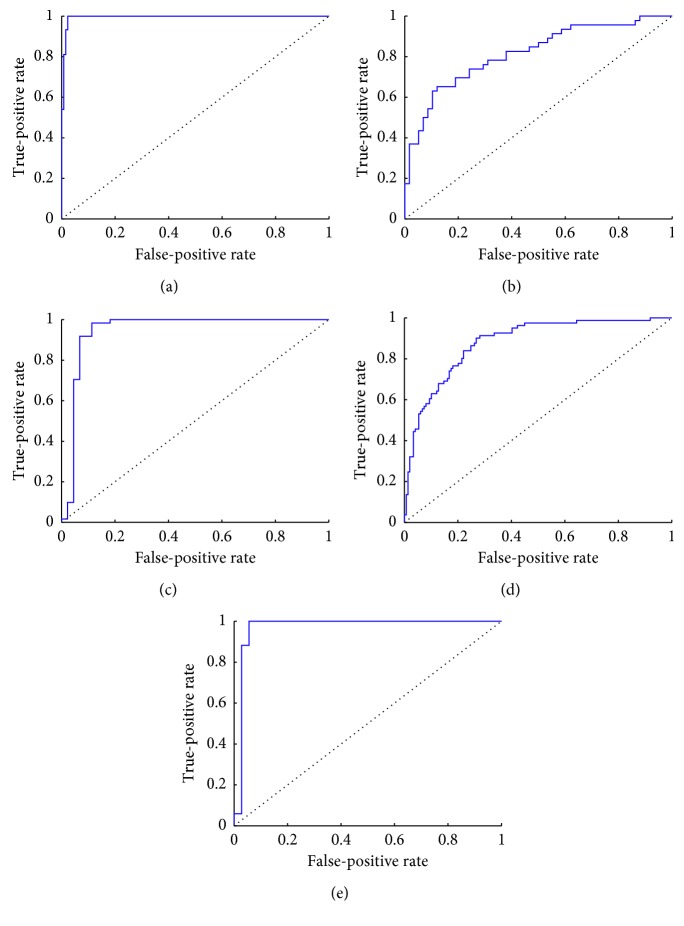
ROCs of Mr^2^DNM that used the optimal feature subsets for five datasets. (a) WBCD. (b) BUPA. (c) IONA. (d) PIMA. (e) VOTE.

The optimal classification accuracy results of the proposed Mr^2^DNM which adopts the reduced feature subsets are summarized in Table 3, where the number of features (NF) in the original dataset, the number of features in the optimal subset (#) obtained by Mr^2^ criteria, the reduction rate of features of the optimal subset to the original one, corresponding feature sequence obtained by Mr^2^ criteria, average accuracy based on 30 independent runs, computational time, and average area under the receiver operator characteristic curve (AUC) for five classification problems are listed. To further prove the effect of Mr^2^ on the DNM classifier, Figure 5 illustrates the influence of used feature size on accuracy and calculation time for classifying five datasets, respectively. It is observed that as the number of features decreases, the accuracy rate changes. Compared with the results that more features are used, a specific subset of features can obtain better accuracy with a lower computational cost. However, too few features will cause the accuracy rate to deteriorate significantly. In addition, the ROCs that can prove the classification quality of classifiers are shown in Figure 6. AUC is the area under ROC, and its range is [0,1] [38]. It means that the classifier can perfectly classify the dataset, when the value of AUC is 1. If the AUC is equal to 0.5, it means the model is a random classifier [39]. According to Table 3, it can be found that Mr^2^DNM obtains high accuracy on WBCD, IONO, and VOTE, and relatively low one on BUPA and PIMA. The low accuracy is caused due to complexity of datasets, and existent literatures also obtain similar results.

**Figure 7:**
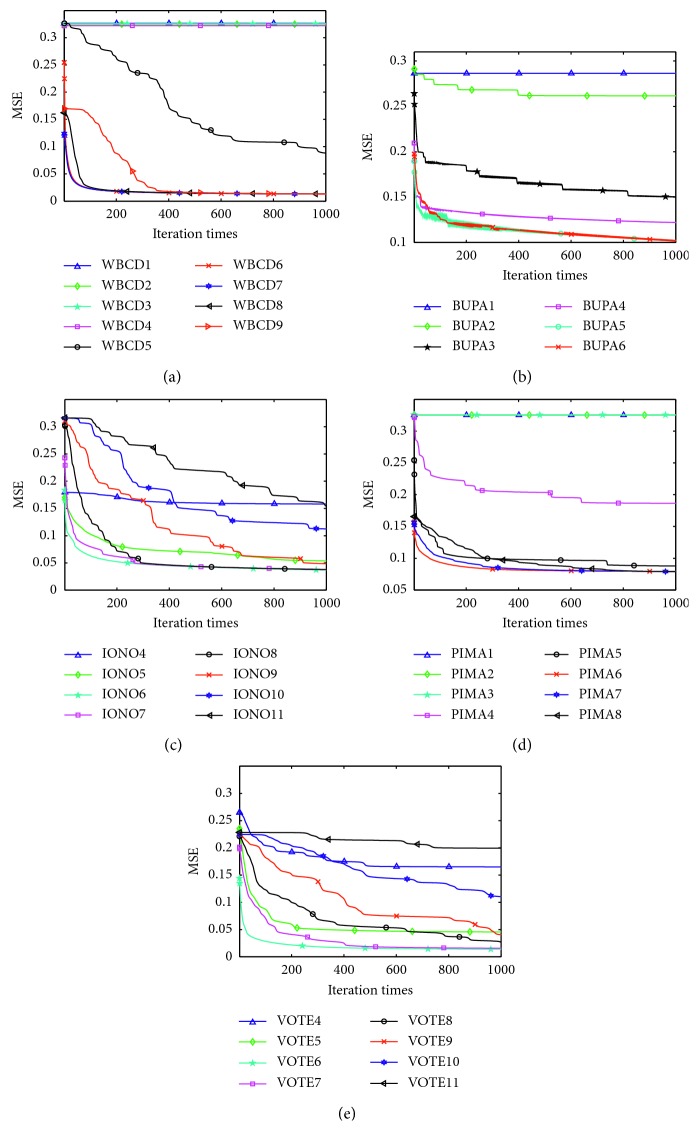
MSE of each feature size for five datasets. (a) WBCD. (b) BUPA. (c) IONA. (d) PIMA. (e) VOTE.

To compare the convergence speed of each feature size, the mean squared error (MSE) of Mr^2^DNM at each iteration is calculated and illustrated in Figure 7, which provides the results of 1000 iterations for five datasets. In Figure 7, the number shown in the legend denotes the feature size. The curves of only eight consecutive subset sizes are shown for IONO and VOTE datasets, which contain the optimal subset size. From Figure 7, it is observed that a better accuracy rate always can be obtained by removing appropriate redundancy features and resulting in a fast convergence speed and a smooth convergence curve. Therefore, Mr^2^ feature selection method is effective for DNM to deal with classification tasks.

**Figure 8:**
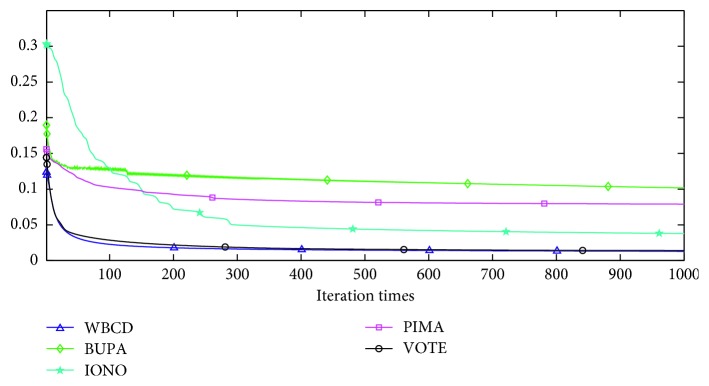
MSE of the used optimal feature sizes for five datasets.

The convergence situations of the five optimal subsets are shown in Figure 8. It is clear that five datasets have all completed their own convergence within 500 iterations. Generally, the reduction of features leads to a lower calculation time. The redundant features are sequentially excluded from the feature subsets so that the classification accuracy changes. However, a reduced feature subset clearly can contribute a better accuracy with a lower calculation cost and faster and smoother convergence situation in comparison with that all features are used. It should be noted that overly small feature size conspicuously reduces the classification accuracy. For the above reasons, Mr^2^DNM is verified to be an optimal compromised method that maximizes the classification accuracy and synchronously minimizes the feature size and calculation time.

Furthermore, the performance of Mr^2^DNM is compared with other six related classification algorithms, including standard back-propagation (Orig) [40], RENN [41], FaLKNR [42], AdaBoost [43], MultiBoost [44], and IE_MLP_ [40].  Table 4 shows the comparative results of the classification accuracy on five benchmark datasets, and the corresponding ranks of performance are listed. The proposed Mr^2^DNM obtains the best accuracy on three classification problems and the average rank (A.Rank) for five classification problems, which is first place among all compared methods. In fact, it can be considered that there is no one algorithm that always outperforms the others on all classification tasks. However, the A.Rank suggests that the performance of the proposed Mr^2^DNM averagely outperforms the other classification techniques.

### 4.3. Simplified Morphology Analysis

#### 4.3.1. Neuron Morphology

**Figure 9:**
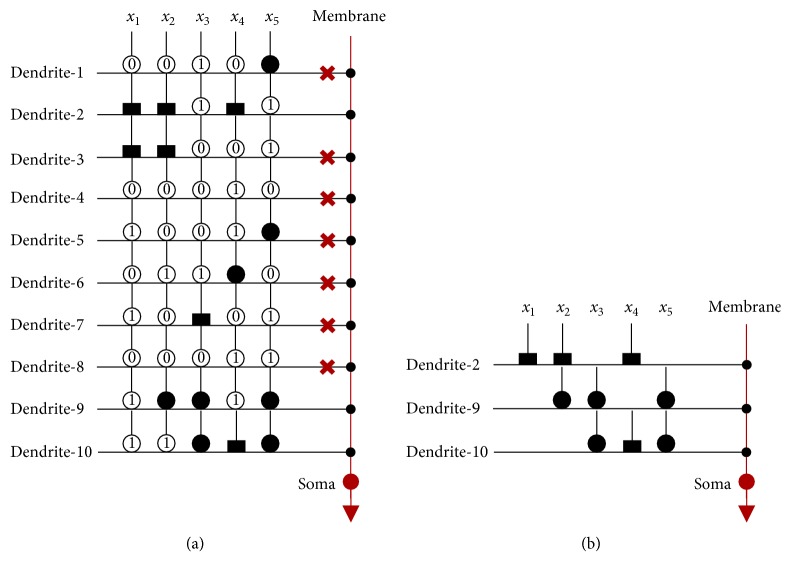
The dendritic morphology of BUPA dataset. (a) After learning. (b) After pruning.

As mentioned above, Mr^2^DNM achieves the internal dimensional reduction via simplifying the morphology to the neuron model during learning. During learning, (1) each attribute has an input (synapse) connection on each dendritic branch; (2) an input connection is defined as one of four connection states whenever a connection action occurs; (3) four connection states are a direct connection (•), an inverse connection (▬), a constant-0 connection (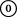), and a constant-1 connection (➀), respectively; (4) the same feature does not necessarily have the same connection type on each dendritic branch; and (5) all the dendritic branches are finally summed in the membrane layer. The internal dimensional reduction is implemented via ignoring the inputs (synapses) which have the constant-1 connection and removing the dendritic branches which have the input of the constant-0 connection states. The neuronal morphology of BUPA as an example is given in Figure 9. Since Mr^2^ is employed as the feature selection, the initial number of the feature is set as 5 at the beginning, which means that DNM reduces the calculation of 10 connection states before training the model. In addition, before training the model, there are 50 synaptic points and 10 dendritic points to perform calculation, as shown in Figure 9(a). After training, the model obtains a simplified morphology which only has 9 synaptic points and 3 dendritic points through the neuron pruning, as shown in Figure 9(b).

#### 4.3.2. Logic Circuits Morphology

**Figure 10:**
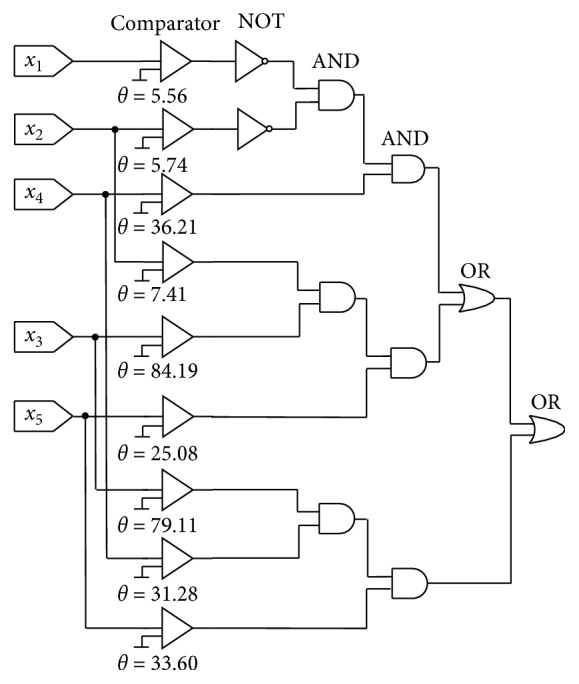
Logic circuit of the simplified morphology of BUPA dataset.

The functions of Mr^2^DNM approximately correspond to the “comparator,” logical “NOT,” “AND,” and “OR” operation, respectively [32, 45]. Thence, the simplified neuron morphology can be replaced by the logic circuits, and the corresponding logic circuits for the BUPA as an example are shown in Figure 10. The comparator of the logic circuit compares the input with the corresponding threshold. If the value of the input exceeds the threshold *θ*, the result outputs 1, and otherwise 0. The final output of the model can be obtained by subsequent logic circuits. The implementation of the simplified model can be realized by the logic circuit in hardware so that the results are easily reproduced while decreasing the computational cost.

## 5. Conclusion

In this paper, a hybrid model (Mr^2^DNM) by considering the feature redundancy and nonlinear interactions in a dendrite tree is used for classifying the practical problems with a low computational cost. The mutual information-based Mr^2^ criterion can cut out redundant features to provide an optimal feature subset for the training of DNM. DNM trained by BP learning algorithm handles major classification work with the plastic mechanism and sigmoid functions. In addition, the simplified morphology of the proposed model obtained by training can be achieved via logic circuits to further decrease cost.

The contribution of study is summarized as follows: (1) an efficient hybrid classification model (Mr^2^DNM) is proposed; (2) the simulation proves that a feature selection method combined with a neuron model can obtain beneficial results; (3) to our knowledge, the hybrid of feature selection method and single neuron model is a research area that still needs to be explored deeply and to provide an inspiring view; and (4) meanwhile, this study advocates others to employ feature selection method to other neural network models for reaching superior classification performance, and it can be expected that such hybridization can avoid the negative impact brought by the redundancy features in the datasets and make the performance of the model fully reflected.

## Figures and Tables

**Table 1:** Datasets used in the experiment.

Dataset	Feature		Sample
	Nominal	Continuous	
WBCD	9	0	683
BUPA	0	7	345
IONO	0	34	351
PIMA	0	8	768
VOTE	16	0	232

**Table 2:** Parameter setting for five datasets.

Dataset	*k*	*k*_soma_	*θ*_soma_	*M*	*η*	No. of iterations	No. of samples	
							Training	Testing
WBCD	1	10	0.5	45	0.01	1000	478	205
BUPA	3	10	0.5	10	0.005	2000	242	103
IONO	3	10	0.5	34	0.001	1000	246	105
PIMA	3	10	0.5	25	0.001	1000	538	230
VOTE	3	10	0.5	30	0.001	1000	162	70

**Table 3:** Performance of the proposed Mr^2^DNM for five datasets.

Dataset	NF	#	Reduction (%)	Optimal feature sequence	Accuracy (%)	Time (×10^3^ s)	AUC
WBCD	9	7	22.22	F2, F6, F1, F7, F5, F3, F8	96.80	54.4	0.9942
BUPA	7	5	28.57	F5, F6, F1, F4, F3	72.66	7.1	0.7458
IONO	34	8	76.47	F5, F1, F8, F4, F3, F28, F7, F14	90.73	24.6	0.9227
PIMA	8	7	12.5	F2, F5, F8, F6, F4, F1, F3	76.80	33.2	0.8198
VOTE	16	6	62.5	F4, F5, F12, F3, F14, F8	96.57	10.2	0.9779

**Table 4:** Average classification accuracy (%) obtained by 30 runs for all compared classifiers.

Dataset		Orig	RENN	FaLKNR	AdaBoost	MultiBoost	IE_MLP_	Mr^2^DNM
WBCD	Accuracy (%)	95.28	96.14	96.28	94.99	95.85	96.62	**96.80**
	Rank	6	4	3	7	5	2	1
BUPA	Accuracy (%)	71.59	71.88	71.01	71.88	71.59	71.59	**72.66**
	Rank	5	2.5	7	2.5	5	5	1
IONO	Accuracy (%)	91.17	86.61	86.61	91.17	**91.74**	89.23	90.73
	Rank	2.5	6.5	6.5	2.5	1	5	4
PIMA	Accuracy (%)	75.39	76.69	75.91	75.26	75.13	**78.07**	76.80
	Rank	5	3	4	6	7	1	2
VOTE	Accuracy (%)	94.71	94.71	96.55	94.48	94.48	95.95	**96.57**
	Rank	4.5	4.5	2	6.5	6.5	3	1
	A.Rank	4.6	4.1	4.5	4.9	4.9	3.2	**1.8**

## Data Availability

The five classification datasets could be downloaded freely at https://archive.ics.uci.edu/ml/index.php.
